# Comparison of Theiler’s Murine Encephalomyelitis Virus Induced Spinal Cord and Peripheral Nerve Lesions Following Intracerebral and Intraspinal Infection

**DOI:** 10.3390/ijms20205134

**Published:** 2019-10-16

**Authors:** Wen Jin, Eva Leitzen, Sandra Goebbels, Klaus-Armin Nave, Wolfgang Baumgärtner, Florian Hansmann

**Affiliations:** 1 Department of Pathology, University of Veterinary Medicine Hannover, 30559 Hannover, Germany; Wen.Jin@tiho-hannover.de (W.J.); Eva.Leitzen@tiho-hannover.de (E.L.); Florian.Hansmann@tiho-hannover.de (F.H.); 2 Center for Systems Neuroscience, 30559 Hannover, Germany; 3 Department of Neurogenetics, Max-Planck-Institute for experimental Medicine, 37075 Göttingen, Germany; SGoebbels@em.mpg.de (S.G.); nave@em.mpg.de (K.-A.N.)

**Keywords:** Theiler’s murine encephalomyelitis virus-induced demyelinating disease, inflammation, intraspinal infection, peripheral nerve lesions

## Abstract

Hallmarks of Theiler’s murine encephalomyelitis virus (TMEV)-induced demyelinating disease (TMEV-IDD) include spinal cord (SC) inflammation, demyelination and axonal damage occurring approximately 5–8 weeks after classical intracerebral (i.c.) infection. The aim of this study was to elucidate the consequences of intraspinal (i.s.) TMEV infection and a direct comparison of classical i.c. and intraspinal infection. Swiss Jim Lambert (SJL)-mice were i.s. infected with the BeAn strain of TMEV. Clinical investigations including a scoring system and rotarod analysis were performed on a regular basis. Necropsies were performed at 3, 7, 14, 28 and 63 days post infection (dpi) following i.s. and at 4, 7, 14, 28, 56, 98, 147 and 196 dpi following i.c. infection. Serial sections of formalin-fixed, paraffin-embedded SC and peripheral nerves (PN) were investigated using hematoxylin and eosin (HE) and immunohistochemistry. I.s. infected mice developed clinical signs and a deterioration of motor coordination approximately 12 weeks earlier than i.c. infected animals. SC inflammation, demyelination and axonal damage occurred approximately 6 weeks earlier in i.s. infected animals. Interestingly, i.s. infected mice developed PN lesions, characterized by vacuolation, inflammation, demyelination and axonal damage, which was not seen following i.c. infection. The i.s. infection model offers the advantage of a significantly earlier onset of clinical signs, inflammatory and demyelinating SC lesions and additionally enables the investigation of virus-mediated PN lesions.

## 1. Introduction

Theiler’s murine encephalomyelitis virus (TMEV), a member of the Picornaviridae family, induces a demyelinating leukomyelitis after intracerebral (i.c.) infection of susceptible mouse strains like Swiss Jim Lambert (SJL) [1,2,3]. Following i.c. infection with a low neurovirulent TMEV strain like BeAn, susceptible mice develop a biphasic disease course consisting of an initial polioencephalitis (early phase) followed by virus persistence and the emergence of demyelinating spinal cord (SC) lesions (late phase), also called TMEV-induced demyelinating disease (TMEV-IDD) [4,5,6]. TMEV-IDD represents a well-established animal model for demyelinating diseases in humans, especially resembling important features of the progressive forms of multiple sclerosis (MS) [6,7,8]. This immune-mediated, inflammatory disease of the central nervous system (CNS) not only constitutes the most frequent cause of non-traumatic neurological disease of young people, but also shows an increasing incidence worldwide [9,10,11]. Treatment possibilities, especially regarding the relapsing-remitting forms of MS, have largely improved over the last years, however, treatment of the progressive form is still unsatisfying and MS remains an incurable disease [12,13,14].

In TMEV-IDD, SC demyelination is most prominent within the thoracic segment, usually occurring after a latency period of around five to eight weeks post i.c. infection [15,16,17]. I.c. infection of resistant mouse strains (e.g., C57BL/6; B6), capable of removing the virus from the CNS, show an acute polioencephalitis but do not develop TMEV-IDD [18,19,20]. Previous investigations demonstrated that switching the infection site from cerebrum to the thoracic SC has far-reaching consequences on the course of the disease [21,22]. Intraspinal (i.s.) TMEV infection resulted in SC inflammation and demyelination in susceptible as well as resistant mice [21,22]. Interestingly, resistant B6 mice showed a fast onset of persistent clinical signs as well as inflammatory and demyelinating SC lesions, comparable to those seen in TMEV-IDD, but also within a markedly shortened time period of about two weeks [22].

However, neither an investigation of i.s. TMEV infection in susceptible mice over an extended period of time nor a direct comparison of i.c. and i.s. TMEV infection in SJL mice has been carried out so far. The hypothesis of the present study was that (i) i.s. infection of SJL mice would result in clinical signs, (ii) establishment of virus persistence as well as (iii) inflammation and demyelination within the SC comparable to lesions following i.c. infection but with a significantly earlier onset.

## 2. Results

### 2.1. Intraspinal TMEV Infection

#### 2.1.1. Clinical Investigation

**Figure 1:**
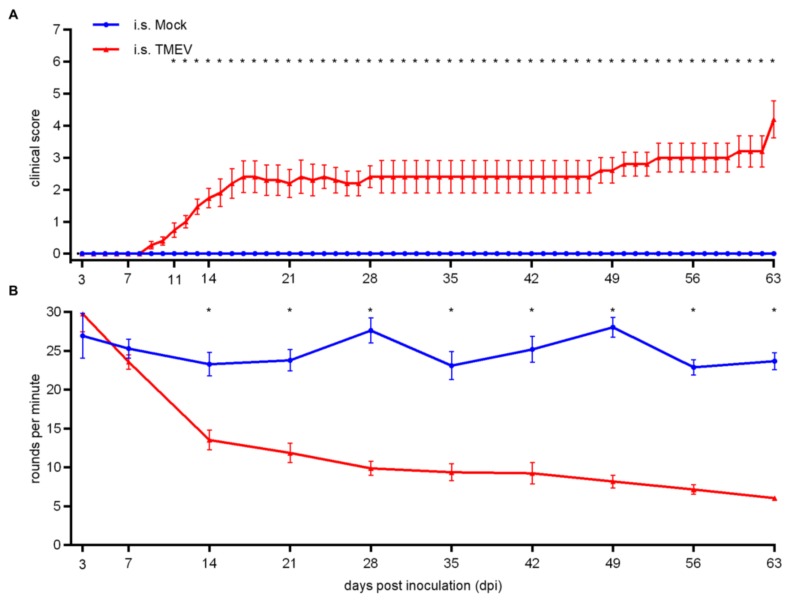
Clinical investigation including a rotarod performance of Swiss Jim Lambert (SJL) mice following intraspinal (i.s.) Theiler’s murine encephalomyelitis virus (TMEV) infection/mock-injection. After 11 days post infection (dpi), TMEV-infected animals showed significantly elevated clinical scores (**A**). Deterioration of motor coordination started at 14 dpi (**B**). Graphs display mean (solid line) and standard error of the mean (SEM). Significant differences between the groups as detected by ANOVA followed by a Mann–Whitney U posthoc test are marked by asterisks (* *p* < 0.05).

Following i.s. TMEV infection, mice showed significant clinical signs starting at 11 days post infection (dpi; Figure 1A). Clinical signs included a shaggy and dull coat, an abnormal posture including a hunched back, reduction of spontaneous locomotion and induced movement, gait abnormalities starting as unilateral weakness and lameness, gradually progressing to severe spinal ataxia and paresis of the hindlimbs. Moreover, TMEV-infected animals showed a deterioration of motor coordination starting at 14 dpi (Figure 1B). Elevated clinical scores as well as the deterioration of rotarod performance were persistent and progressing until 63 dpi.

#### 2.1.2. Spinal Cord Lesions

**Figure 2:**
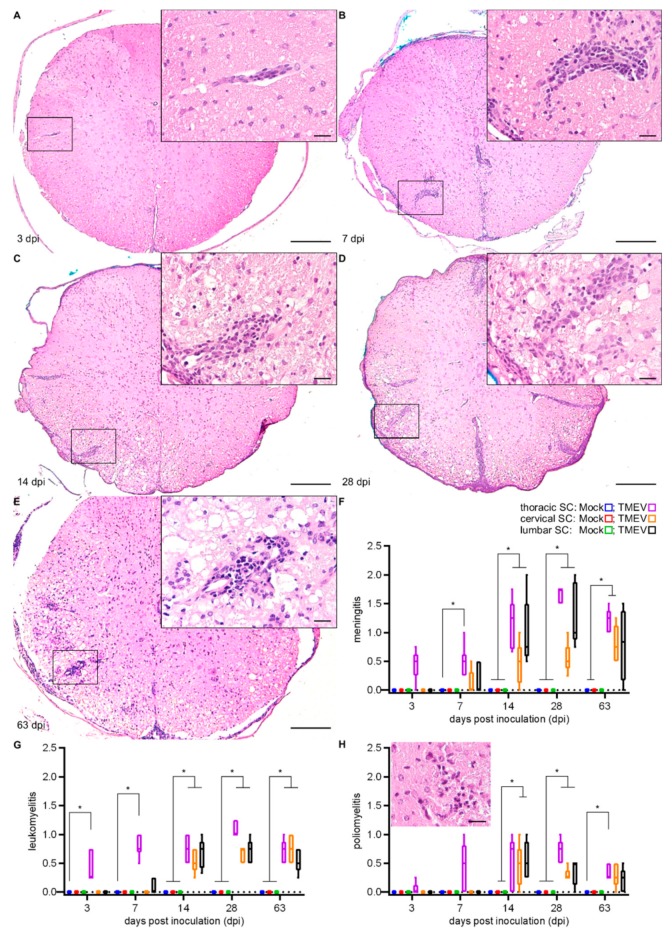
Histopathological changes following i.s. TMEV infection at 3 (**A**), 7 (**B**), 14 (**C**), 28 (**D**) and 63 (**E**) dpi in the thoracic SC (injection site). Lesions consisted of meningeal/perivascular lymphocyte infiltration (**A**–**E**) and demyelination (**C**–**E**, indicated by the loss of eosinophilia) within the white matter. Meningitis (**F**), leukomyelitis (**G**) and poliomyelitis (**H**) showed a rostral and caudal dissemination starting from the injection site. Poliomyelitis was most frequently located in the ventral horns (insert in H). Box-and-whisker plots show median and quartiles. Significant differences between the groups as detected by a Mann–Whitney U-test are indicated by asterisks (* *p* < 0.05). Hematoxylin and eosin, bars represent 200 μm in the overviews and 20 µm in the inserts.

Inflammatory lesions (Figure 2A–E), characterized by lymphocytes and microglia/macrophages, in infected animals were initially found within the white matter of the thoracic SC segment (injection site) at 3 dpi. Significant infiltration of lymphocytes and macrophages within the meninges were firstly detected at 7 dpi, followed by a combination of meningitis and leukomyelitis, affecting all three SC segments at 14 dpi (Figure 2F,G). In addition, poliomyelitis, most frequently located in the ventral horns, was detected in the cervical and lumbar segments at 14 dpi while all investigated segments were affected at 28 dpi (Figure 2H). 

**Figure 3:**
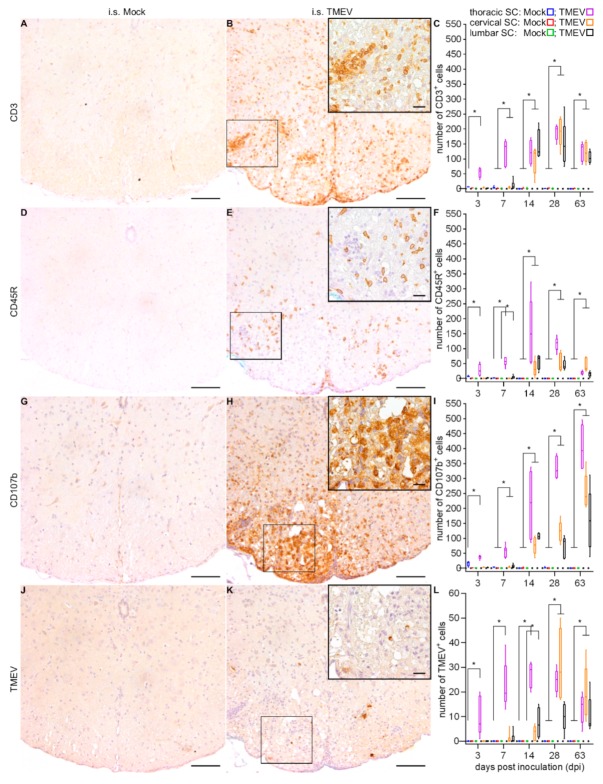
Immunophenotyping of inflammatory cells and quantification of TMEV positive cells within the SC following i.s. mock-injection (at 14 dpi; **A**,**D**,**G**,**J**) and TMEV infection (at 14 dpi; **B**,**E**,**H**,**K**). Statistical analysis revealed significantly increased numbers of CD3^+^ (T lymphocytes), CD45R^+^ (B lymphocytes) and CD107b^+^ (microglia/macrophages) cells (**C**,**F**,**I**), as well as a spread of virus protein (**L**) following i.s. TMEV infection. Box-and-whisker plots show median and quartiles. Significant differences between the groups as detected by a Mann–Whitney U-test are indicated by asterisks (* *p* < 0.05). Bars represent 100 μm in the overviews and 20 µm in the inserts.

Associated with gray matter inflammation, neuronal degeneration was occasionally detected. Immunohistochemical phenotyping of inflammatory cells revealed CD3^+^ T lymphocytes (Figure 3A–C), CD45R^+^ B lymphocytes (Figure 3D–F) and CD107b^+^ microglia/macrophages (Figure 3G–I) with microglia/macrophages and T lymphocytes being the predominant cell types. In accordance with the results from the evaluation of HE sections, inflammation was initially centered around the injection site with subsequent antero- and retrograde dissemination until 63 dpi. Viral protein was detected in the thoracic SC at all investigated time points, first being restricted to the injection site, followed by caudal spread to the lumbar segment (14 dpi). From 28 dpi until 63 dpi, virus protein wasdetected in all SC segments (Figure 3J–L).

**Figure 4:**
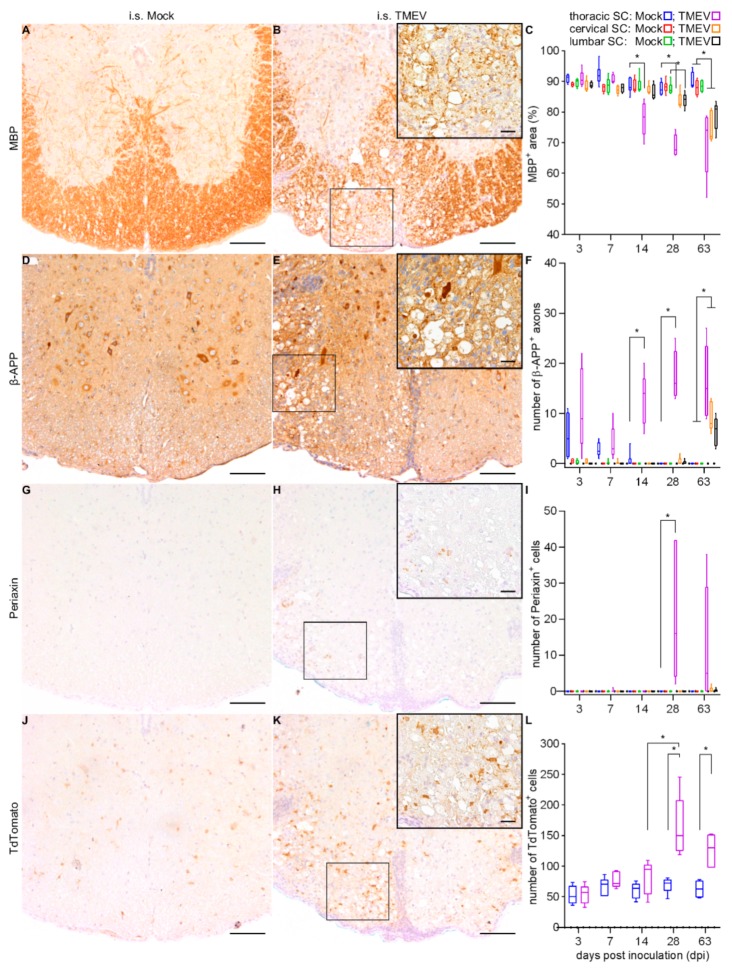
Detection of demyelination (myelin basic protein—MBP) and axonal damage (β-APP) at 14 dpi within the thoracic SC following i.s. mock-injection (**A**,**D**) and TMEV infection (**B**,**E**). Characterization of regenerative attempts indicated by infiltration of periaxin^+^ Schwann cells and TdTomato^+^ glial cells of the NG2 lineage at 28 dpi within the thoracic SC following i.s. Mock-injection (**G**,**J**) and TMEV infection (**H**,**K**). Statistical analysis revealed significant demyelination starting at 14 dpi (**C**) and an increasing number of damaged axons (**F**), as well as a subsequent (28 dpi) increase in periaxin^+^ and TdTomato^+^ cells, interpreted as remyelinating attempt (**I**,**L**). Box-and-whisker plots show median and quartiles. Significant differences between the groups as detected by a Mann–Whitney U-test are indicated by asterisks (* *p* < 0.05). Bars represent 100 μm in the overviews and 20 µm in the inserts.

Associated with the spatial and temporal distribution of inflammation and TMEV protein, demyelination, as detected by a reduction of myelin basic protein (MBP) labeled white matter area, was observed within the thoracic SC at 14, 28 and 63 dpi, within lumbar SC at 28 and 63 dpi, and within cervical SC at 63 dpi (Figure 4A–C). Axonal damage as detected by β-APP around the injection site of TMEV-infected animals at 14, 28 and 63 dpi, affecting lumbar and cervical SC at a late time point (63 dpi; Figure 4D–F). The occurrence of periaxin^+^ Schwann cells and TdTomato^+^ NG2 lineage cells were observed in the thoracic SC of TMEV-infected animals at late time points (28 and 63 dpi). At 28 dpi, the number of Schwann cells (Figure 4G–I) and NG2 lineage cells (Figure 4J–L) was significantly increased in the thoracic SC while mock-injected animals did not show any periaxin^+^ cells. 

#### 2.1.3. Peripheral Nerve Lesions

**Figure 5:**
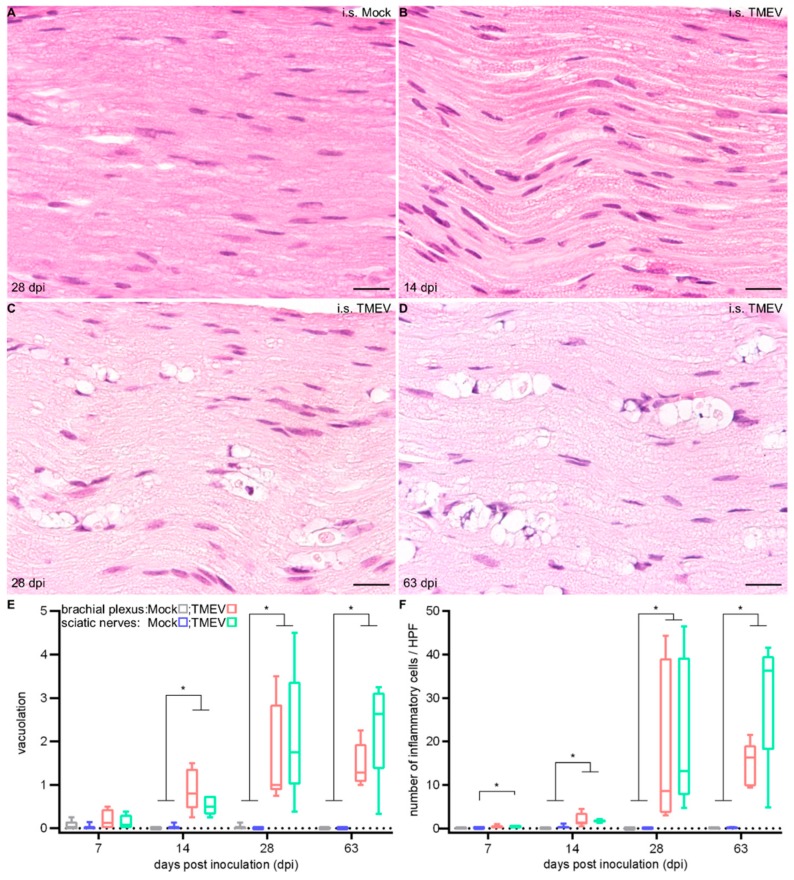
Histopathogical examination of the peripheral nerve (PN) at 28 days post i.s. mock-injection (**A**) and at 14 (**B**), 28 (**C**) and 63 (**D**) days post i.s. TMEV infection. PN showed vacuolation (**E**) and inflammation (**F**). Graphs display the weighted average of left and right brachial plexus or sciatic nerves, respectively. Box-and-whisker plots show median and quartiles. Significant differences between the groups as detected by a Mann–Whitney U-test are indicated by asterisks (* *p* < 0.05). Hematoxylin and eosin, bars represent 20 μm.

An increased cellularity, starting at 7 dpi as well as a vacuolation of peripheral nerve fibers, starting at 14 dpi, were observed (Figure 5). 

**Figure 6:**
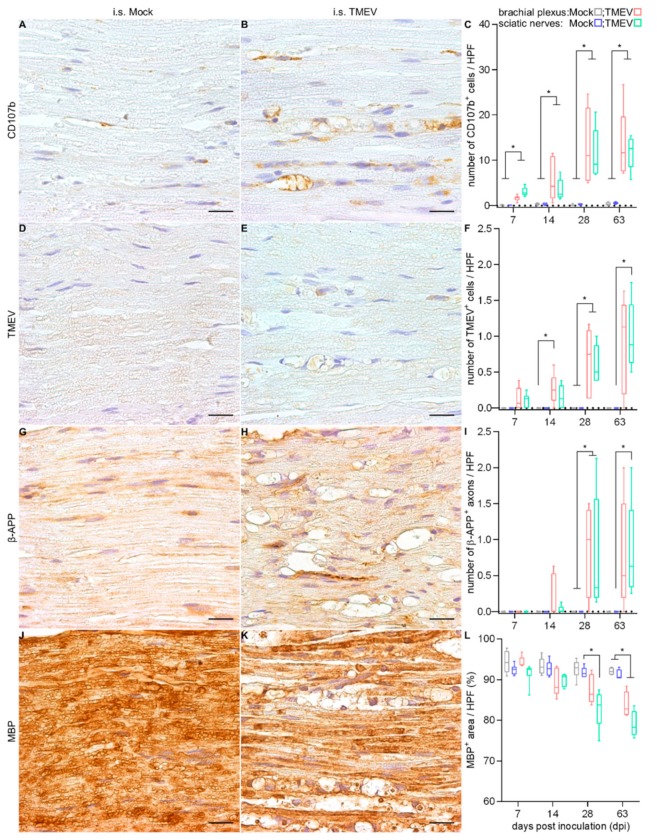
Detection of macrophages and myelinophages, TMEV, axonal damage and demyelination at 28 dpi in PN following i.s. mock-injection (**A**,**D**,**G**,**J**) and TMEV infection (**B**,**E**,**H**,**K**). Statistical analysis revealed a significant increase in the number of CD107b^+^ macrophages (**C**) at all investigated time points. Virus protein was firstly detected at 7 dpi, while significant differences started at 14 dpi (**F**). Significant axonal damage (**I**), as well as demyelination (**L**) following i.s. TMEV infection were initially seen at 28 dpi. Graphs show the average of left and right brachial plexus or sciatic nerves, respectively. Box-and-whisker plots show median and quartiles. Significant differences between the groups as detected by a Mann–Whitney U-test are indicated by asterisks (* *p* < 0.05). Bars represent 20 μm.

Immunohistochemical phenotyping of inflammatory cells revealed an increased number of CD107b^+^ macrophages including myelinophages (Figure 6A–C), whereas only few CD3^+^ T lymphocytes were detected. TMEV^+^ cells were found in the brachial plexus at 14 and 28 dpi, as well as in sciatic nerves at 28 and 63 dpi (Figure 6D–F). The number of β-APP^+^ axons was significantly increased in TMEV-infected animals at 28 (brachial plexus and sciatic nerves) and 63 dpi (sciatic nerves; Figure 6G–I). In TMEV-infected animals, a significant and progressive demyelination was detected starting at 28 dpi within sciatic nerves (Figure 6J–L).

### 2.2. Intracerebral TMEV Infection

#### 2.2.1. Clinical Investigation

**Figure 7:**
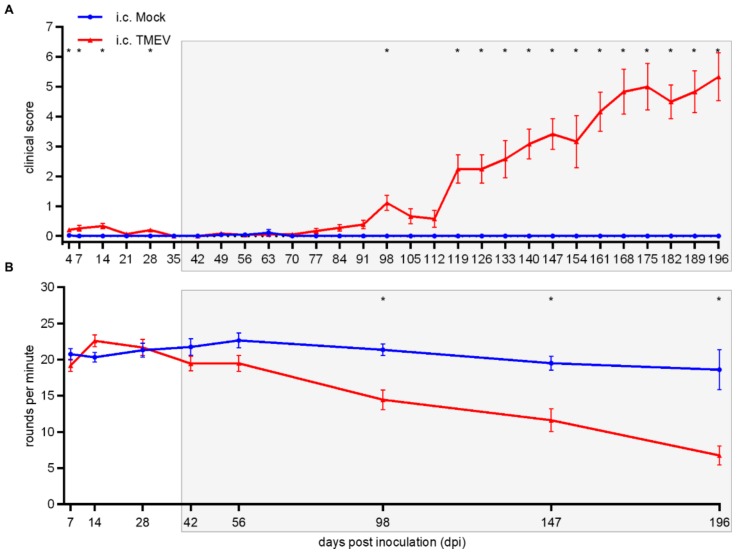
Clinical investigation and rotarod performance test of SJL mice following intracerebral (i.c.) TMEV infection/mock-injection. At 4, 7, 14, 28 and 98 dpi and 119–196 dpi, TMEV-infected animals showed significantly elevated clinical scores (**A**). Deterioration of motor coordination was detected starting at 98 dpi (**B**). Graphs display mean (solid line) and standard error of the mean (SEM). Significant differences between the groups as detected by ANOVA followed by Mann–Whitney U posthoc tests are marked by asterisks (* *p* < 0.05).

During the late phase of TMEV-IDD following i.c. TMEV infection, clinical signs were firstly noticed at 98 dpi (Figure 7A), along with a deterioration of motor coordination (Figure 7B). Elevated clinical scores as well as deterioration of rotarod performance were persistent and progressive until the end of the investigation period (196 dpi).

#### 2.2.2. Spinal Cord Lesions

**Figure 8:**
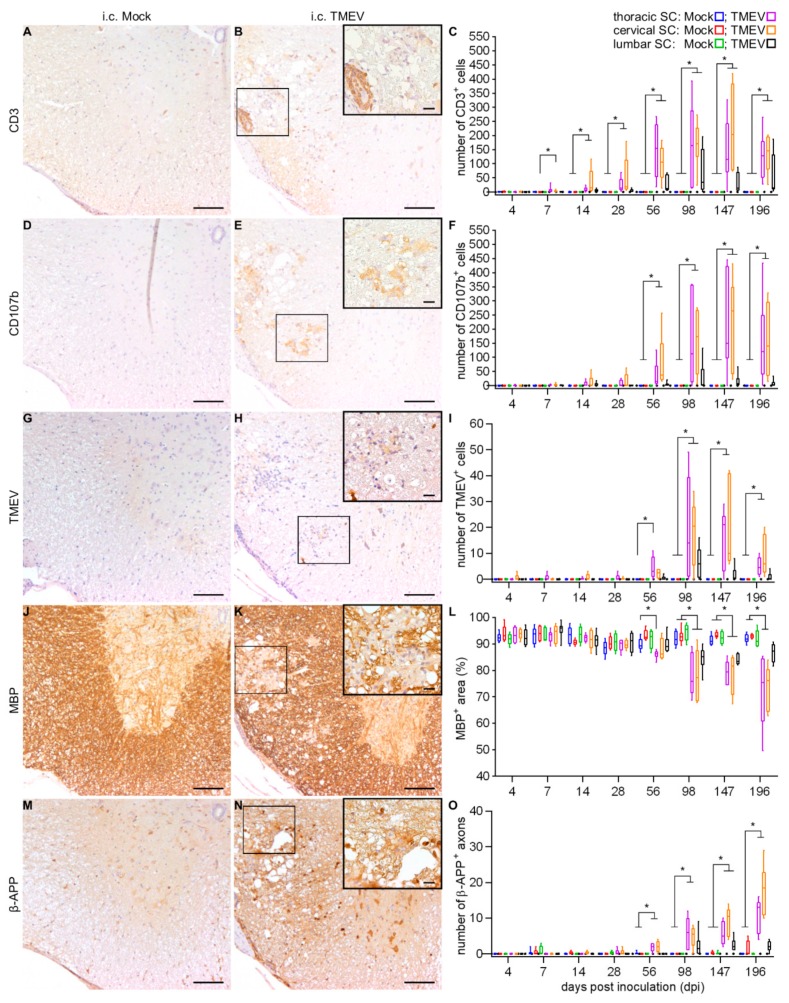
Detection and quantification of the SC lesion following i.c. TMEV infection/mock-injection. Immunophenotyping of inflammatory cells as well as detection of virus protein, demyelination and axonal damage at 56 dpi within the thoracic SC following i.c. mock-injection (**A**,**D**,**G**,**J**,**M**) and TMEV infection (**B**,**E**,**H**,**K**,**N**). Statistical analysis revealed a significantly increasing number of CD3^+^ T lymphocytes followed by an increased number of CD107b^+^ microglia/macrophages (**C**,**F**). The presence of virus protein (**I**), as well as progressive demyelination (**L**) and axonal damage (**O**) starting at 56 dpi were detected following i.c. TMEV infection. Box-and-whisker plots show median and quartiles. Significant differences between the groups as detected by a Mann–Whitney U-test are indicated by asterisks (* *p* < 0.05). Bars represent 100 μm in the overviews and 20 µm in the inserts.

Immunohistochemical phenotyping of inflammatory cells in i.c. TMEV-infected animals revealed increasing numbers of inflammatory cells, mainly composed of CD3^+^ T lymphocytes; starting at 7 dpi within the cervical SC (Figure 8A–C) and CD107b^+^ microglia/macrophages starting at 56 dpi within the cervical and thoracic SC (Figure 8D–F), along with a limited number of CD45R^+^ B lymphocytes. Demyelination (Figure 8J–L) and axonal damage (Figure 8M–O), accompanied by the presence of TMEV (Figure 8G–I), were firstly observed at 56 dpi, and progressed until the end of the investigation period. An elevated number of periaxin^+^ cells was detected in the cervical SC at 196 dpi.

#### 2.2.3. Peripheral Nerve Lesions

**Figure 9:**
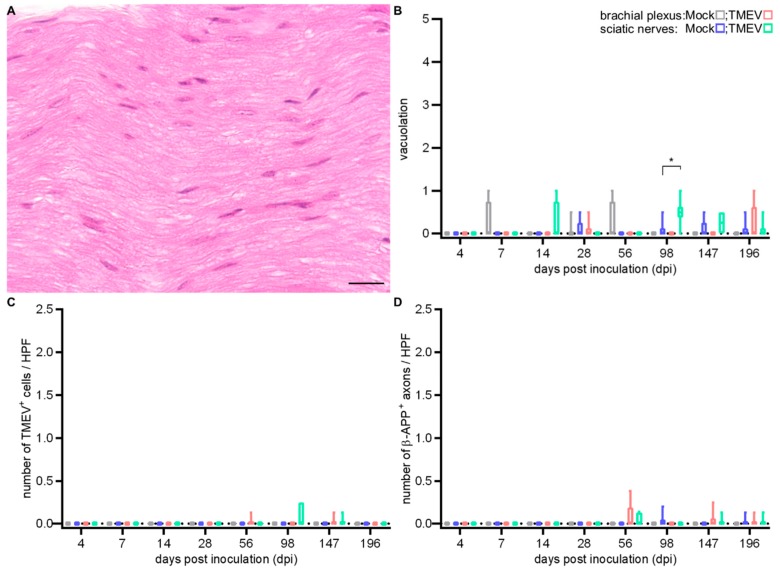
Light microscopical examination following i.c. TMEV infection revealed no lesions in sciatic nerves at 56 dpi (**A**). A mild vacuolation of PN was observed at 98 dpi in sciatic nerves. (**B**), while virus protein (**C**) as well as axonal damage (**D**) were occasionally observed. Graphs display the weighted average of left and right brachial plexus or sciatic nerves, respectively. Box-and-whisker plots show median and quartiles. Significant differences between the groups as detected by a Mann–Whitney U-test are indicated by asterisks (* *p* < 0.05). Bars represent 20 μm.

Within the peripheral nerve (PN) of i.c. TMEV-infected animals, only a mild degree of vacuolation was noticed (Figure 9B). Virus protein (Figure 9C) and axonal damage (Figure 9D) were occasionally seen at 56, 98 and 147 dpi.

## 3. Discussion

Different routes of experimental TMEV infection have been investigated since its discovery in the 1930s [23,24]. Besides the probably most common i.c. infection, additional infection routes including intranasal, intramuscular (gastrocnemius and tongue), intraneural (sciatic and hypoglossal nerve), intravenous, intraperitoneal and intrafootpad were described [18,24,25,26,27,28]. The i.c. infection of susceptible mouse strains with low neurovirulent TMEV strains of the Theiler’s original (TO) subgroup represents a well-established animal model for demyelinating diseases in the CNS, especially for the progressive forms of MS [8,29,30]. Hallmarks of TMEV-IDD include inflammation, demyelination, axonal damage and astrogliosis predominantly in the ventral part of the SC [7]. Mechanisms leading to demyelination in TMEV-IDD include TMEV-induced oligodendrocyte apoptosis [16] as well as a T lymphocyte driven antiviral immune response initially targeting viral antigens followed by the development of a type IV hypersensitivity during the chronic phase [31,32]. Additional mechanisms leading to the development of autoimmune processes against oligodendrocytes in TMEV-IDD include epitope spreading and a molecular mimicry between viral and myelin epitopes [7,33,34,35,36,37,38]. It has been shown that i.s. TMEV infection with Daniel’s (DA) strains can be used to study the local, early events of TMEV-induced demyelination. This model offers the advantage of a fast onset of demyelinating events, in combination with a precise lesion site [21]. In the present study, using the BeAn strain in SJL mice, i.s. TMEV-infected animals showed a comparable early onset of clinical signs (11 dpi) and a deterioration of motor coordination starting at 14 dpi, compared to i.c. TMEV-infected mice where clinical signs started at 98 dpi. Our data indicate that shifting the infection site to the SC results in an approximately 12 weeks earlier onset of clinical signs compared to i.c. infection. However, besides the time shift the clinical course of Theiler’s murine encephalomyelitis in SJL mice following i.s. (starting at 3 dpi) and i.c. (starting at 42 dpi) infection was very similar (Figure 7 indicated by gray background). The accelerated clinical deterioration is most likely a direct consequence of the i.s. TMEV infection, as the initial early phase of polioencephalitis with the need of a subsequent establishment of virus persistence in the SC is bypassed.

**Figure 10:**
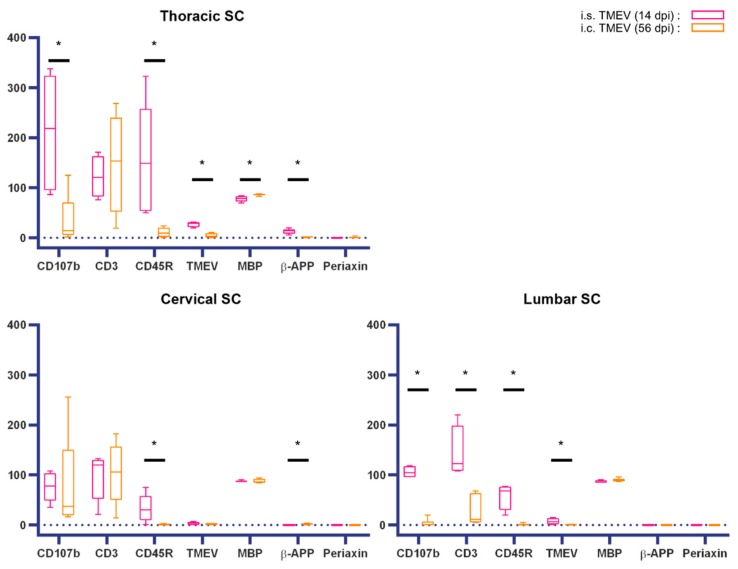
Comparison of a spinal cord (SC) lesion in intraspinal (i.s.) versus intracerebral (i.c.) TMEV-infected mice at 14 or 56 days post infection (dpi), respectively. Data are given for each spinal cord (SC) segment separately including numbers of immunolabeled cells for CD107b (microglia/macrophages), CD3 (T lymphocytes), CD45R (B lymphocytes), TMEV (Theiler’s murine encephalomyelitis virus) and periaxin (Schwann cells) and immunolabeled axons for β-APP (beta-amyloid precursor protein; axonal damage). Furthermore, MBP (myelin basic protein) was used for the quantification of demyelination and data are presented as percentage of total white matter area. Data are presented as box-and-whisker plots showing median and quartiles. Significant differences between the i.s. and i.c. infection models as detected by a Mann–Whitney U-test are indicated by asterisks (* *p* < 0.05).

Accordingly, inflammatory and degenerative changes within the SC following i.s. infection were detected after a markedly shortened time span compared to the classical i.c. infection model. A direct comparison of the thoracic SC, representing the most severely affected segment after i.c. TMEV infection [5,39,40] and the injection site in the thoracic SC used in this study, reveals that inflammatory infiltrates, especially CD107b^+^ microglia/macrophages, appear very early after TMEV infection. I.c. TMEV-infected animals showed an inflammatory reaction in the spinal cord accompanied by demyelination and axonal damage delayed in time compared to i.s. infected mice. A considerable comparability, especially regarding inflammation, presence of virus protein and demyelination between both infection routes was identified at 14 dpi (i.s.) and 56 dpi (i.c.), emphasizing the advantage of i.s. infection leading to a markedly shortened time span of around 6 weeks for the development of SC lesions. However, in TMEV-IDD, i.c. infected mice show a leukomyelitis while i.s. infected animals showed a leuko- (starting at 3 dpi) and poliomyelitis (starting at 14 dpi, Figure 2), occasionally associated with neuronal degeneration. Gray matter inflammation associated with neuronal alterations may have additionally contributed to the observed earlier onset of clinical signs as well as the development of PN lesions in i.s. infected mice. Statistical analysis shows that nearly all investigated parameters were more pronounced after i.s. infection, which might be a result of the local virus injection at this site. This conclusion is supported by the observation that differences between other segments are not that pronounced at this time point (Figure 10).

Besides the circumvention of the early phase following i.c. infection, inflammatory and degenerative changes following i.s. infection start in the thoracic SC. Afterwards, inflammation disseminated into the cervical and lumbar SC, whereas after i.c. infection inflammation and demyelination followed an unidirectional, caudally directed propagation involving the lumbar SC at comparably late time points. However, the distribution of inflammatory lesions and the appearance of demyelinated foci centered in the ventral white matter areas were comparable between both models. Moreover, the qualitative composition of inflammatory cell types and the consistent presence of virus protein, indicating the establishment of a virus persistence, accompanied by chronic progressive demyelination within the SC and a deterioration of motor function until the end of the investigation period were similar.

In the present study, TdTomato was used as an inducible fluorescent marker of cells of the NG2 lineage [41]. The detected cells not only include NG2-expressing cells, but also the matured stages of these progenitor cells, as well as pericytes [42,43,44]. An increased number of both periaxin^+^ Schwann cells and TdTomato^+^ glial cells was found at 28 dpi after i.s. TMEV infection. The occurrence of Schwann cells and the accumulation of TdTomato^+^ glial cells around demyelinating lesions indicated remyelinating attempts [22,45,46,47]. However, regenerative attempts were detected much earlier in i.s. compared to i.c. infected animals where NG2^+^ cells were detected as early as 42 dpi [48] and periaxin^+^ cells even later (196 dpi). Accordingly, the i.s. infection model could offer the possibility to investigate remyelinating events after virus induced demyelination over an extended time period, not only using the advantage of a shortened time span until lesion onset but also eliminating interferences caused by age-related degenerative changes (e.g., spontaneous myopathy or lymphoma [49,50]). Another advantage of i.s. infection is the distinct lesion site of SC infection. This enables to distinguish precisely between the initial, early lesion site and the secondary sites of demyelination after viral spread. Further investigation can take the advantage of this i.s. TMEV infection model to gain more insight into the long-term consequences of de- and remyelinating events within the CNS. Moreover, the pathological hallmarks of the i.s. infection model largely correspond to those seen in TMEV-IDD. Our results indicate that the i.s. and i.c. TMEV infection model show SC lesions sharing disease characteristics with MS (e.g., white matter demyelination, inflammation and axonal damage) [22,51,52,53,54]. However, further investigations are required to figure out whether i.s. infection also mimics other features seen in MS and TMEV-IDD, like the blood–brain barrier impairment [55].

Interestingly, inflammatory and degenerative changes within the SC were followed by significant alterations within PN after i.s. TMEV infection, which has never been detected to this extent after i.c. TMEV infection. I.s. infected SJL mice revealed a mononuclear inflammation and vacuolation in PN, which was also seen in i.s. infected C57BL/6 mice [22]. The occurrence of TMEV in PN indicates that TMEV is capable of invading the PN. Unlike in C57BL/6, the presence of the virus protein following i.s. TMEV infection was not an occasional event [22]. Numbers of positive cells increased over time, indicating an ongoing spread or maybe a virus proliferation within PN. Bidirectional axoplasmic flow represents a possible route for virus trafficking within the nervous system. This fits previous studies where TMEV protein was found to travel to the SC after intraneural-injection into the sciatic nerve [27]. The presence of macrophages, being the predominant inflammatory cell type, also raises the question whether infected macrophages are a crucial component for virus spread to the peripheral nervous system (PNS). It is known that axonopathy in TMEV-IDD is the result of both, outside-in and inside-out mechanisms [56,57,58], which may also play a role in PN lesions. Whether inflammatory reactions within the PN represent a forwarded event originating in the SC, or if they constitute an independent phenomenon as a result of a compartmentalized immune response between PNS and CNS has to be determined in future studies [59,60,61].

## 4. Materials and Methods

### 4.1. Experimental Animals, Virus Infection and Tissue Processing

For i.s. injection, randomized groups of 4–6, female, double heterozygous SJL.NG2CreERT2^+/−^×Rosa26.floxed.stop-tdTomato^+/−^ mice were housed in a microisolator cage system (Tecniplast, Hohenpeiβenberg, Germany). Mice were generated using conventional backcrossing over 10 generations on SJL/J background (Jackson Laboratories, Bar Harbor, ME, USA). Animals had free access to drinking water and food. For induction of TdTomato expression within NG2^+^ (nerve/glial antigen 2) cells, mice continuously received a tamoxifen-containing special diet (TD55125, 400 mg/kg tamoxifen citrate; Envigo, Indianapolis, IN, USA) starting 1 week prior to i.s. TMEV infection/mock-injection [22,62]. At four to five weeks of age mice were anesthetized using an intraperitoneal administration of ketamine (100 mg/kg; ketamine 10%, Bela-pharm, Vechta, Germany) and medetomidine (0.5 mg/kg; Domitor^®^, Orion Pharma, Espoo, Finland) in combination with analgesic treatment (tramadol, 15 mg/kg, Tramadol-Ratiopharm^®^; Ratiopharm, Ulm, Germany and Carprofen, 4 mg/kg, Rimadyl^®^; Pfizer, New York City, NY, USA) as previously described [22]. Mice were placed in a small animal stereotaxic instrument (TSE Systems, Bad Homburg, Germany) and a hemilaminectomy and durectomy at the level of the 10^th^ thoracic vertebra were performed. Each animal received a stereotaxic injection of either 4.56 × 10^3^ plaque-forming units (PFU) of the BeAn strain of TMEV or an equivalent volume of cell culture supernatant (mock) into the ventral part of the SC using a 10-μL syringe (Hamilton, Bonaduz, Switzerland) with a previously stretched glass capillary (Drummond Microcaps^®^, Sigma-Aldrich, Seelze, Germany). Injections were performed at an angle of 10° and a depth of 1 mm. After injection the needle was left in place for 1 min to allow the injected fluid to diffuse. During surgical procedures, eyes were covered with ointment (Bepanthen^®^ Augen and Nasensalbe; Bayer AG, Leverkusen, Germany). Moreover, animals received an additional analgesic treatment (tramadol, 1 mg/mL) via drinking water until 3 days post surgery. Perfusions (0.01 M phosphate buffered saline; 3.75 mL/min) followed by necropsy were performed at 3, 7, 14, 28 and 63 dpi. For direct comparison of i.s. and i.c. infection, additional serial sections of murine SC and peripheral nerves (PN) from a previous study were used [48]. In this study, groups of six, five-week-old female SJL/JCrl mice (Charles River Laboratories, Sulzfeld, Germany) were i.c. infected with the BeAn strain (1.63 × 10^6^ PFU per animal) of TMEV or cell culture supernatant [48]. Necropsies were performed at 4, 7, 14, 28, 56, 98, 147 and 196 dpi.

All tissue samples were fixed in 10% formalin followed by embedding in paraffin wax (FFPE). For SJL/JCrl mice, complete SC segments including vertebral bones were decalcified in ethylenediaminetetraacetic acid for 48 h. SC of the thoracic, including the injection site, cervical and lumbar segments, as well as PN including proximal and median aspects of left and right brachial plexus and sciatic nerves were cut on a microtome (Leica RM 2035; Leica Instruments GmbH, Nuβloch, Germany). Transversal serial sections (2–3 µm) of the SC and longitudinal serial sections of the PN were prepared for hematoxylin and eosin (HE) staining as well as immunohistochemistry (IHC).

### 4.2. Clinical Investigations

Clinical investigations were performed daily (after i.s. infection) or once a week (after i.c. infection). For clinical investigations, a semiquantitative scoring system was applied. The scoring system included three categories: external appearance and posture (0–3), behavior and activity (0–3), as well as gait (0–4) [22]. The final score was calculated as a sum of all three categories. In addition, an accelerated rotarod test (RotaRod Treadmill; TSE Technical and Scientific Equipment, Bad Homburg, Germany) was performed as previously described [22,63]. For each animal and time point, the mean value of three consecutive runs was calculated.

### 4.3. Immunohistochemistry

For IHC, sections of the cervical, thoracic and lumbar SC segments as well as the PN were stained with primary antibodies (Table 1) targeting virus protein (TMEV), CD3 (T lymphocytes), CD45R (B lymphocytes), CD107b (microglia/macrophages), myelin basic protein (MBP; myelin), β-amyloid precursor protein (β-APP; axonal damage), periaxin (Schwann cells) and dsRed (TdTomato) as previously described [15,22,63,64]. For antigen visualization, the avidin-biotin-peroxidase complex method (Vectastain ABC Kit; Vector Laboratories, Burlingame, CA, USA) and 3,3′-diaminobenzidine-tetrahydrochloride (Sigma-Aldrich, St. Louis, MO, USA) treatment, followed by counterstaining with Mayer′s hemalaun were used.

### 4.4. Histological Examination

HE stained transverse sections of the thoracic, cervical and lumbar SC segments were evaluated using a semiquantitative score quantifying meningitis and perivascular inflammation in the gray (poliomyelitis) as well as in the white matter (leukomyelitis). The inflammatory score ranged from 0 to 3 (0: normal; 1: scattered infiltrates; 2: 2–3 layers of inflammatory cells and 3: more than 3 layers of inflammatory cells). IHC targeting CD3, CD107b, CD45R, TMEV, β-APP and periaxin within white matter of SC of each SC segment was evaluated by counting of positive cells or axons, respectively. In addition, Tdtomato-positive glial cells were quantified within the thoracic segment. Quantification of MBP-positive area was performed using analySIS^®^ 3.2 software (SOFT Imaging System; Olympus, Münster, Germany). Slides were digitalized, white matter was manually outlined as the regions of interest (ROI), and a threshold value adjusted [15]. MBP-positive area was calculated as a percentage of the outlined ROI.

For PN, vacuolation was quantified within the most severely affected high power field (HPF; 400×) using a semi-quantitative grading scale ranging from 0 to 5 (0: no vacuoles; 1: ≤3 vacuoles; 2: <10% affected; 3: 10–20% affected; 4: 20–30% affected and 5: 30–40% affected) and inflammatory cells were counted on HE stained slides. For IHC targeting CD3, CD107b, TMEV and β-APP within nerve fibers, positive cells or axons were counted, respectively. Data is shown as average number of positive cells or axons per HPF. Quantification of MBP-positive area of PN was performed in analogy to SC sections using the most severely affected HPF per nerve. Data is shown as the average value of left and right brachial plexus or sciatic nerves, respectively.

### 4.5. Statistical Analysis

Graphs were created using GraphPad Prism for Windows version 8.0 (GraphPad Software, La Jolla, CA, USA). Statistical analysis was performed using SPSS for Windows version 25 (IBM^®^ SPSS^®^ Statistics, SPSS Inc., Chicago, IL, USA). Data were analyzed using ANOVA followed by a Mann–Whitney U posthoc tests between TMEV-infected and mock-injected animals. A *p*-value < 0.05 was accepted as statistically significant.

### 4.6. Ethics Statement

All animal experiments were conducted in accordance with the German Animal Welfare Law and were approved by Niedersächsisches Landesamt für Verbraucherschutz und Lebensmittelsicherheit (LAVES, Oldenburg, Germany; permission numbers: 33.12-42502-04-15/1996 (29 December 2015) and 33.9-32502-04-07/1292 (14 June 2007)). 

## 5. Conclusions

The present study shows that i.s. infection of SJL mice resulted in the emergence of neurological deficits in the context of inflammatory and demyelinating SC lesions, comparable to those seen after i.c. infection. Moreover, there was also evidence for the establishment of virus persistence following i.s. infection. The i.s. infection model offered the advantage of investigating precise temporal and local virus induced SC lesions over a long time period, which could be especially interesting regarding the elucidation of remyelinating attempts in the presence of infectious virus. Moreover, the emergence of a peripheral neuropathy after i.s. infection could offer potential as a new animal model for studying pathogen-induced human PNS demyelinating diseases like the Guillain-Barré syndrome.

## Figures and Tables

**Table 1:** Antibodies and related reagents employed in immunohistochemistry.

1st Antibody			Pre-Treatment	Blocking Serum	2nd Antibody
Antigen, Target	Product Name	Dilution			
CD3, T lymphocytes	Dako A0452	1:250	microwave, citrate buffer	goat	goat anti-rabbit
CD107b, microglia/macrophages	BioRad MCA2293	1:400	microwave, citrate buffer	rabbit	rabbit anti-rat
CD45R, B lymphocytes	BD Bioscience 553085	1:1000	microwave, citrate buffer	-	-
Capsid protein 1 (VP1), TMEV	[65]	1:2000	-	goat	goat anti-rabbit
Myelin basic protein (MBP), myelin	Chemicon AB980 GeneTex GTX32733	1:500 1:250	- -	goat	goat anti-rabbit
Beta-amyloid precursor protein (β-APP), axonal damage	Merck/Millipore MAB348	1:2000	microwave, citrate buffer	goat	goat anti-mouse
Periaxin, Schwann cells	Sigma HPA001868	1:5000	microwave, citrate buffer	goat	goat anti-rabbit
LivingColor DsRed, Tdtomato	Takara/Clontech 632496	1:500	microwave, citrate buffer	goat	goat anti-rabbit

## Abbreviations

β-APP	β-amyloid precursor protein
CNS	central nervous system
Crl	Charles River Laboratories
DA strain	Daniel’s strain
FFPE	formalin-fixed paraffin-embedded tissue
HE	hematoxylin and eosin
HPF	high power field
I.c.	Intracerebral
I.s.	Intraspinal
MBP	myelin basic protein
MS	Multiple sclerosis
NG2	nerve/glial antigen 2
PFU	plaque forming units
PN	peripheral nerves
PNS	peripheral nervous system
ROI	region of interest
SJL	Swiss Jim Lambert
SC	spinal cord
TMEV	Theiler′s murine encephalomyelitis virus
TMEV-IDD	Theiler´s murine encephalomyelitis virus-induced demyelinating disease
